# DEM Study of the Motion Characteristics of Rice Particles in the Indented Cylinder Separator

**DOI:** 10.3390/s23010285

**Published:** 2022-12-27

**Authors:** Xinzhi Yu, Xuesong Jiang, Haiyang Gu, Fei Shen

**Affiliations:** 1 College of Mechanical and Electronic Engineering, Nanjing Forestry University, Nanjing 210037, China; 2 School of Food Science and Engineering, Nanjing University of Finance and Economics, Nanjing 210023, China

**Keywords:** discrete element method, escape angle, indented cylinder separator, Kullback-Leibler divergence, trough position, separation efficiency

## Abstract

The precise separation of rice particles is an important step in rice processing. In this paper, discrete element simulations of the motion of rice particles of different integrity in an indented cylinder separator were carried out using numerical simulation methods. The effects of single factors (cylinder rotation rate, cylinder axial inclination angle, and collection trough inclination angle) on the motion trajectories of particles are investigated and the probability distribution functions of particles are obtained. The statistical method of Kullback-Leibler divergence is used to quantitatively evaluate the differences in the probability distribution functions of the escape angles of particles of different degrees of integrity. The purpose of this paper is to determine the optimum parameters for an indent cylinder separator by understanding the material cylinder separating process from particle scale and to provide a basis for the numerical design of a grain particle cylinder separators.

## 1. Introduction

Rice is one of the most valuable biological resources on which humankind depends, and its processed form is loved by many and has a large market. The market price of rice is influenced by the size of the grains. Processed white rice can be divided into whole fine rice, broken rice, and small broken rice according to its length, with the price of whole fine rice being two or even three times higher than that of broken rice. Therefore, during the rice production and processing process, rice grains should be sorted and broken, and impurity particles should be collected to improve the quality of rice and thus its market price and competitiveness [1].

At present, rice sorting equipment can be divided into mechanical sorting and optical sorting according to different principles; mechanical sorting can be divided into flat sorting and rotation sorting; optical sorting is mainly the use of photoelectric converters and other sensors for color selection [2]. In the modern rice processing industry, mechanical sorting is often used as a pre-selection for optical sorting to remove a significant portion of the broken rice particles and reduce the burden of optical sorting. In mechanical sorting, as the difference in grain size between whole rice and broken rice is mainly in the length, indented cylinder separators make it possible to screen the particles more accurately according to their length. At the same time, Indented cylinder separators are widely used due to their simple structure, ease of operation, and ease of maintenance [3].

In the field of indented cylinder separator research, a series of articles have been published to explore the optimum test parameters based on principle analysis and actual test results [4,5,6]. At present, most of the research focuses on establishing the relationship between separation efficiency and the structural and manipulative parameters of the cylinder, such as indent size, cylinder speed, cylinder axial tilt angle, collection trough position, collection trough inclination, etc. An J. [7] analyzed the various factors affecting the separation quality in terms of the structure of the separation machine and the separation principle and came up with the main structural factors affecting the separation quality: the shape of the indent, the axial inclination angle of the cylinder, the inclination angle of the collecting trough, the length of the trough baffle of the collecting trough; the cylinder speed is the main manipulative influence. Seo J. et al. [8] conducted separating experiments for different varieties of kernels using three indent radii and collection trough inclination angles to generalize the separation efficiency and provide a reference for experimental data on the selection and design of indented cylinder separators. Kim M.H. et al. [9] used a four-factor, four-level orthogonal rotary combination separating test and found that the shape of the indent (teardrop and hemispherical) did not have a significant effect on the separating efficiency and that the size of indent should be slightly larger than the length of the broken rice to ensure separating efficiency. Optimizing the rice grain separation process requires a clear understanding of the mixing and separation pattern of rice grains within the indented cylinder separators. However, using experimental methods to understand the complex forces on rice particles during their movement within the cylinder is very difficult. Researchers have therefore introduced high-speed photography, mechanical vision, and image processing techniques into the study of separating machines. Buus O.T. et al. [10] used high-speed cameras to extract the trajectory of rice grains during the separating process and invert the escape angle by obtaining information on the position of the grains, achieving a breakthrough in the traditional “black box” test. Liao et al. [11] applied particle tracer technology (PTV) and image processing to investigate the effect of drum speed and filling rate on the separation of particles within the drum and applied shape indices to quantitatively characterize the degree of segregation. However, the problem with the image approach is that only two-dimensional information can be obtained, whereas the actual separating process is three-dimensional, and the overlap between grains makes it more difficult to obtain accurate data.

To solve the problem of incomplete information on a two-dimensional plane caused by graphic processing techniques and to better understand the mixing and separation patterns of materials in the cylinder, researchers have introduced the discrete unit method and numerical simulation into the study of particle sorting. In a study of similar equipment in cylinder sorting machines, Markauskas D. et al. [12] used DEM coupled with SPH (smoothed particle hydrodynamics) to simulate the sorting of plastic waste in a rotating cylinder and investigated the effects of drum speed, feed rate, and number of lifting bars on sorting efficiency. Xie Q. et al. [13] found that the drum speed mainly affects the mixing behavior of the particles in the cylinder, while the structure, height, number, and shape of the lifting bars mainly affect the contact area between the particles and the cylinder walls.

As can be seen, most of the existing studies have focused on the optimization of equipment performance and impurity separation, and less research has been carried out on the analysis of rice particle sorting and material movement patterns in the drum [14]. To our knowledge, reports on the application of DEM in the separation process of indented cylinder separators are extremely sparse. In this paper, a discrete meta-simulation of the motion of rice particles of different integrity in the indented cylinder separator is carried out by means of numerical simulation [15]. The influence of single factors (cylinder rotation speed, cylinder inclination angle, and collection trough inclination) [16] on the trajectory of particles with different degrees of integrity is investigated, and the probability distribution functions of particles with different degrees of integrity are obtained. The statistical method of KL (Kullback-Leibler divergence) distance was used to quantitatively evaluate the differences in the probability distribution functions of the escape angles of particles with different degrees of integrity [17,18,19].

The purpose of this paper is to determine the optimum parameters for an indented cylinder separator by understanding the characteristics of the movement of irregular particles such as rice within the cylinder at the particle scale and mastering the separation pattern of rice particles. Clarifying the movement of rice particles within the cylinder has far-reaching implications for the parametric design of drum separators, not only saving time and material costs in drum design but also providing a basis for the numerical design of indentation cylinder separators. The characteristics of the movement of rice particles inside the drum can also be extended to the rest of the research on drum equipment such as ball mills.

## 2. Simulation Model Building

### 2.1. Cylinder Separation Machine Model

**Figure 1:**
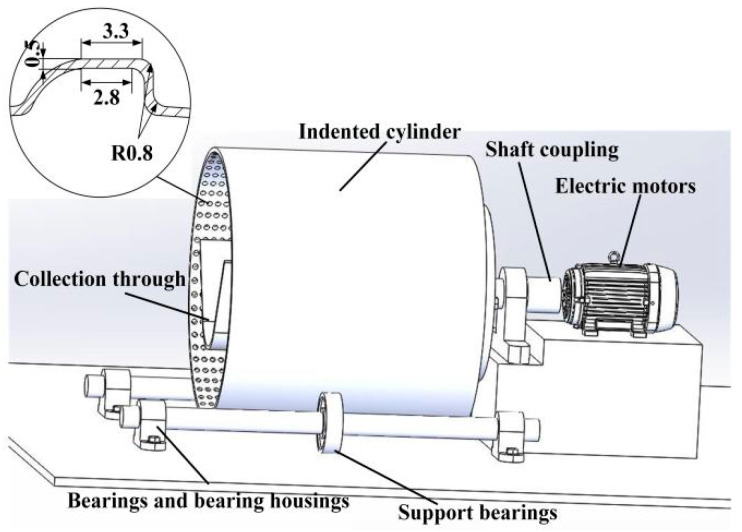
The structure of the cylinder separator.

The entire structure of the cylinder separator is shown in Figure 1. It consists of four main parts, the cylinder body with indents used to separate the particles with different degrees of integrity, the collection trough which collects the broken particles [20], the support for the cylinder, and the motor which transmits the power. The drum model has a length of 300 mm and a radius of 150 mm.

The rice particles entering the cylinder rise under the action of friction between the particles, the particles and the cylinder, and centrifugal inertia forces [21]. At the same time, the short rice particles are selected and contained by the indents on the inner surface of the cylinder and lifted to a higher position. After reaching a certain height, the short particles are released from the indents and fall into the collection trough, thus achieving the separation of long and short particles [22].

### 2.2. Rice Model

In this paper, the japonica rice Dongnong 429 cultivated by Northeast Agricultural University was selected as a prototype for white rice with a milling degree of 7% and a moisture content of 14%. To simplify the rice grain model, it can usually be regarded as an ellipsoid. The present discrete element modelling of rice particles with different degrees of completeness was carried out using EDEM 2018 software [23].

**Figure 2:**
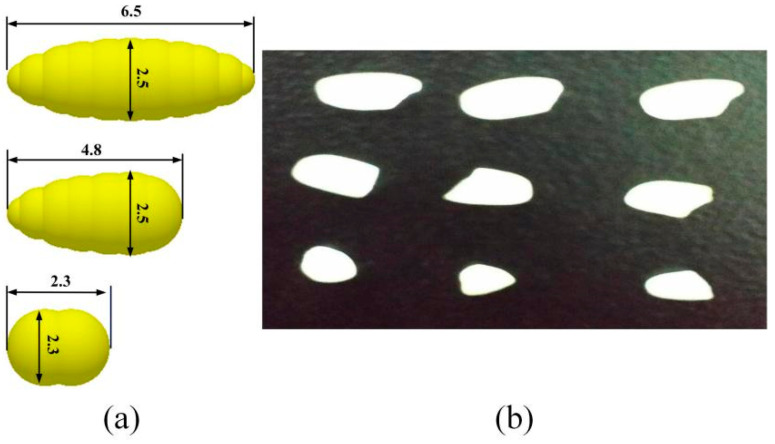
(**a**) Discrete element model of rice particles; (**b**) rice in kind.

The discrete element model is shown in Figure 2. In order to improve the similarity between simulation and reality, the particle models used in the simulation are model groups with a normal distribution of sizes centered on these three rice particle models.

## 3. Mathematical Model

### 3.1. Solid Phase Control Equations in Simulation

In discrete element theory, the linear and rotational motion of the particles obey Newton’s second law. Before the calculation, the contact between the particles and the particles and between the particles and the wall is firstly determined, then the change of force between the particles can be obtained from Newton’s second law, and then the change in the displacement of the particles can be obtained from the change of force. Finally, the state of the entire particle population is determined through continuous iterative calculations [24]. The particle motion control equations are as follows.

(1) $$m_{i}\frac{d\upsilon_{i}}{dt}=\sum f_{i}$$

(2) $$I_{i}\frac{d\omega_{i}}{dt}=\sum M_{i}$$

where $m_{i}$ is the particle mass, kg. t is the calculation time, s. $\upsilon_{i}$ is the particle velocity, m/s. $f_{i}$ is the force acting on the particle, N. $I_{i}$ is the particle rotational inertia, kg·m^2^. $\omega_{i}$ is the particle angular velocity, rad/s. $M_{i}$ is the moment acting on the particle, N/s. Subscripts i indicate different particles.

### 3.2. Analysis of the Dynamics of Particle Movement in Cylinder

**Figure 3:**
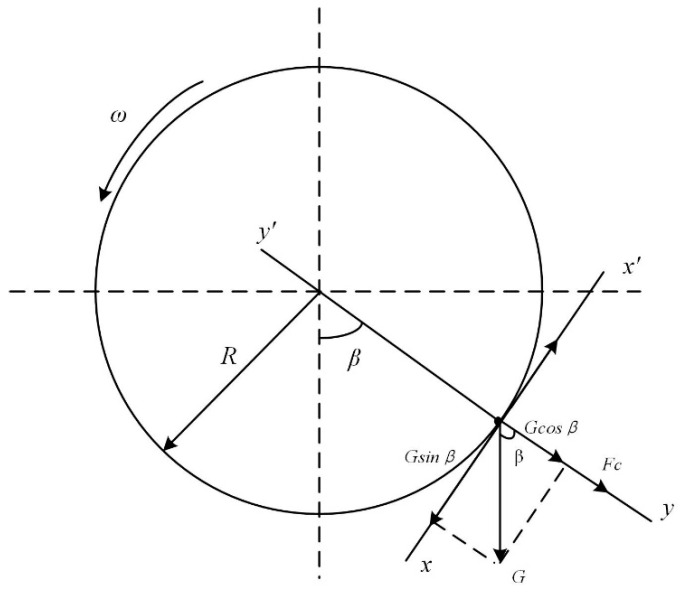
Force analysis of particles in the separator.

During the rotation of the cylinder, too fast a speed can easily lead to the material being subjected to excessive centrifugal force and failing to fall from the indents, while too slow a speed cannot ensure that the material is lifted to a certain height. A reasonable rotational speed is required to separate the broken rice particles entering the indents. The material is mainly subjected to gravity, friction, and centrifugal force during the ascent (Figure 3). The gravity force G is decomposed into 2 forces Gsinβ along the tangential axis $x-x^{\prime }$ and Gcosβ along the normal axis $y-y^{\prime }$. The frictional force $F_{n}$ is then provided by the combined centrifugal force $F_{c}$ and Gcosβ.

The critical state, where the forces of upward and downward movement of the particles reach equilibrium at the maximum cylinder speed $n_{\max}$, can be calculated from Equation (3):

(3) $$G\sin\beta =f(G\cos\beta +F_{c})$$

where G is the gravity, N. β is the angle of detachment of the material, (°). f is the friction factor between the material and the wall of the cylinder. $F_{c}$ is the centrifugal force, N. At this point, the centrifugal force is:

(4) $$F_{c}=\frac{m\upsilon_{n}^{2}}{R}=\frac{mR\pi^{2}n^{2}}{900}$$

where m is the mass of the particle, kg. R is the radius of the cylinder, m. $\upsilon_{n}$ is the linear velocity of the particle movement, m/s. n is the speed of the cylinder, r/min. Combining Equations (3) and (4) gives:

(5) $$n<n_{\max}=\frac{30}{\pi }\sqrt{\frac{g}{R}}$$

According to Equation (5), since the radius of the drum used is 150 mm, the critical speed of this cylinder separator $n_{\max}$ = 77 r/min, $\omega_{\max}$ = 8.1 rad/s. Therefore, 30~70% of the critical drum speed was used as the test parameter in this study.

### 3.3. Kullback–Leibler Divergence

The Kullback-Leibler divergence is based on a probability distribution, and its ability to measure data objects for which geometric distance is difficult to measure is one of its most significant breakthroughs. Assuming that interval D is a continuous interval and are two different probability density functions, respectively, the KL distance of the discrete random variable is shown in Equation (6).

(6) $$D(\rho_{i}\|\rho_{j})=\sum_{x\in D}\rho_{i}(x)\log_{2}\frac{\rho_{i}(x)}{\rho_{j}(x)}$$

The calculation of similarity based on KL scatter requires three steps: smoothing, symmetry correction, and distance-to-similarity conversion [25,26].

(1) Smoothing

To ensure the applicability of the Kullback-Leibler divergence, i.e., to ensure that ρ(x)>0, a smoothing process is required, as shown in Equation (7). where 0<δ<1; after the smoothing process, the inaccuracy tends to 0 when the value of δ tends to 0.

(7) $$\overset{\wedge }{\rho }(x)=\rho (x)+\delta$$

(2) Symmetry correction

From Equation (6), the KL scatter has a completely non-symmetrical nature. Therefore, a symmetrical correction needs to be made when calculating the distance between two items by correcting the KL scatter to the KL distance and using Equation (8) for the calculation of the KL distance between two items.

(8) $$D_{s}(i,j)=(D(\rho_{i}\|\rho_{j})+D(\rho_{j}\|\rho_{i}))/2$$

(3) Distance to similarity conversion

The KL distance-based item similarity calculation obtained from the KL distance of the discrete random variable in Equation (6) is shown in Equation (9).

(9) $$KL(i,j)=sim_{KL}(i,j)=\frac{1}{1+D_{s}(i,j)}$$

From the above equation, the smaller the KL distance, the higher the similarity between the two items, and the larger the KL distance, the lower the similarity.

## 4. Parameter Settings

A total of 40,000 rice particles were generated in the simulation, with 50% intact particles and 25% of each size of broken rice particles. The simulation was carried out for a total of 80 s with a time step of 1 × 10^−5^ s [27]. The existing rice particle physical properties parameters were referred to in this study, and the specific values are shown in Table 1.

## 5. Results and Analysis

After the cylinder was started, the crushed rice particles changed from resting to moving, with large fluctuations. To avoid bringing in disturbing factors in the analysis, the relevant data were counted after the crushed rice particles reached a steady state of t = 10 s.

### 5.1. Model Validation

**Figure 4:**
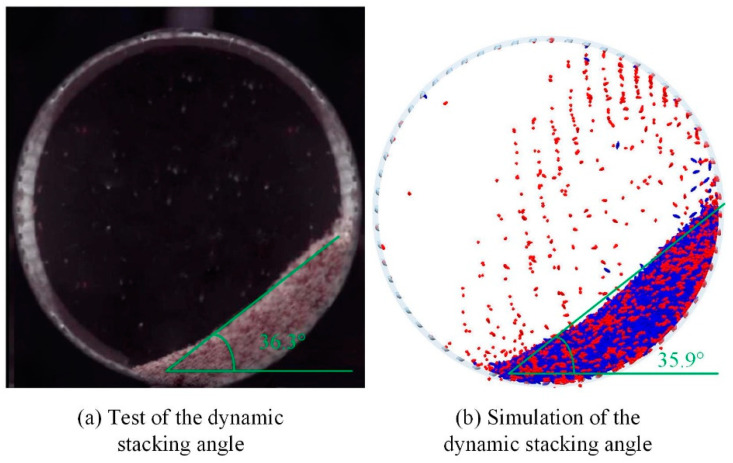
(**a**) Dynamic stacking angle of the experimental model. (**b**) Dynamic stacking angle of the discrete element model.

In this paper, the rotation rate of 30–70% critical speed is selected for comparing the simulation and test dynamic accumulation angle, and the rotation rate is the ratio of the actual to the critical rotational speed. When the whole system reaches a steady state, the dynamic accumulation image of the simulation and the test and the processed figure are obtained, as shown in Figure 4.

Figure 4 shows the dynamic stacking angles between the actual test model and the simulation model at 50% critical speed. It is clear from Figure 4 that the dynamic particle stacking angle of the actual test model is 36.3° and that of the simulated model is 35.9° at 50% critical speed, an error of only 1.11%, verifying the accuracy of the constructed discrete element model.

**Figure 5:**
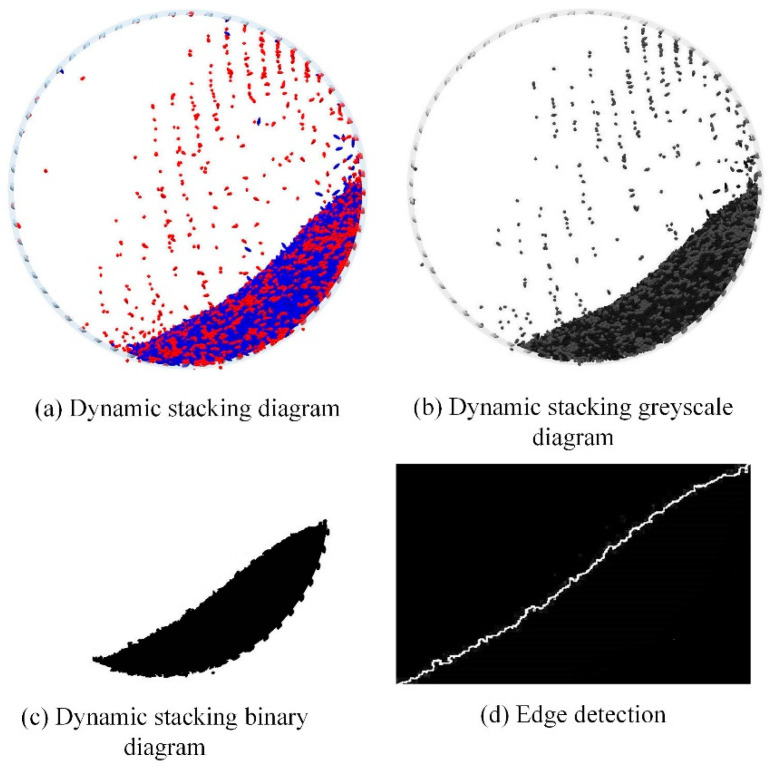
Edge extraction process (**a**) Dynamic stacking diagram. (**b**) Dynamic stacking greyscale diagram. (**c**) Dynamic stacking binary diagram. (**d**) Edge detection.

**Figure 6:**
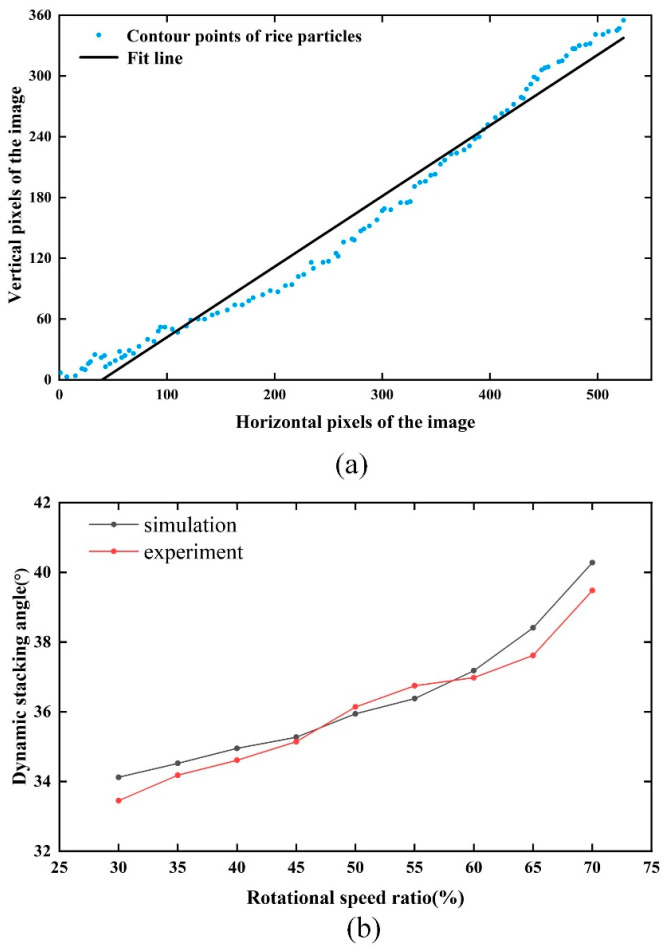
(**a**) Numerical measurement of dynamic stacking angle by image processing; (**b**) dynamic stacking angle as a function of the rotational speed ratio.

Due to the crescent-shaped accumulation of rice particles driven by the drum and the uneven surface of the accumulation layer, it is very difficult to make a virtual straight line of dynamic stacking angles by hand. For quantitative comparisons, accurate and fast measurements avoid human variation and image processing techniques are used to obtain dynamic stacking angles. Dynamic stacking angles were extracted by image processing techniques, with steps shown in Figure 5. Images to be measured were read using the software and displayed in a grayscale plot. Then the binarization was performed, and the sobel operator was applied to detect the image with edges. The stacking angle size was obtained by linear fitting to the acquired edge curves. In Figure 6a, the intersection angle α between the fitted line and the horizontal line is the particle dynamic stacking angle of the corresponding rotational speed.

We can conclude from Figure 6b that the dynamic stacking angle increases with the rotation rate, and the simulation results agree with the actual test results, but the values are slightly different. In the actual test, the cylinder will vibrate slightly on the drive device, and the rice grain size is more dispersed. These factors will lead to the difference between the test and the simulation results. Thus, this paper proves the reliability of the selected model from both qualitative and quantitative aspects.

### 5.2. Velocity Field Distribution in the Indented Cylinder Separator

The distribution of the velocity field in the mixing zone is a direct reflection of the movement of the rice grains in the cylinder.

**Figure 7:**
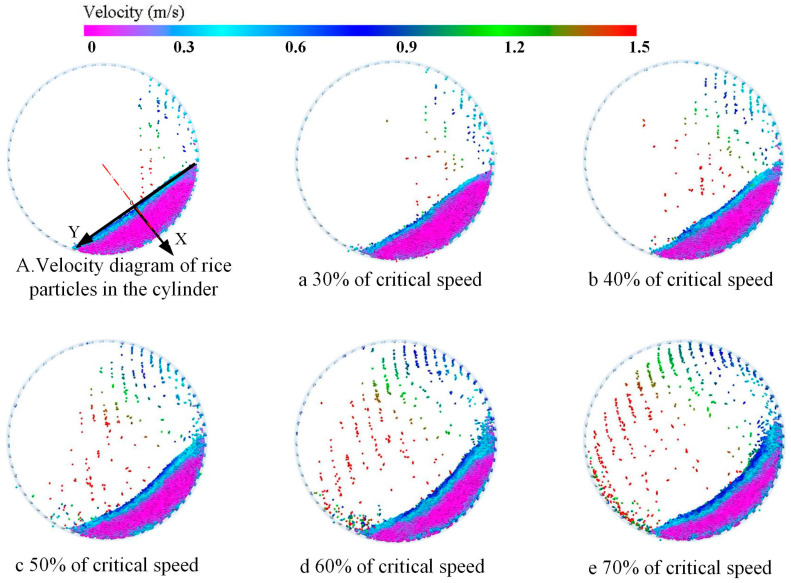
(**A**) Velocity diagram of rice particles in the cylinder. (**a**–**e**) Velocity field distribution at 30%, 40%, 50%, 60%, 70% critical speed.

From Figure 7A, the movement of the rice particles in the indented cylinder separator can be divided into three areas: the free layer near the flow surface, the intersection layer in the mixing center, and the hysteresis layer near the cylinder wall. The free layer is the area of rapid particle flow. After the particles in the cylinder have reached a steady state, the particles in this area are subjected to their own gravity and flow rapidly, with an avalanche of particles falling in the direction of velocity along the free flow surface towards the bottom of the cylinder. When the particles in the intersection layer are distributed near the free layer, the velocity direction of the particles remains the same as that of the particles in the free layer. When the particles are distributed near the hysteresis layer, the velocity direction is the same as that of the particles in the hysteresis layer. At the same time, the displacement of the particles in the center of the zone is less variable than in the other zones, and the particles rotate around the center of the zone. The particles in the hysteresis layer are driven by the cylinder wall and inter-particle shear and move upwards along the wall in the opposite direction to the particles in the free layer. From the above analysis, it can be concluded that the particles in the free and hysteresis layers move at higher speeds and undergo greater displacement changes.

**Figure 8:**
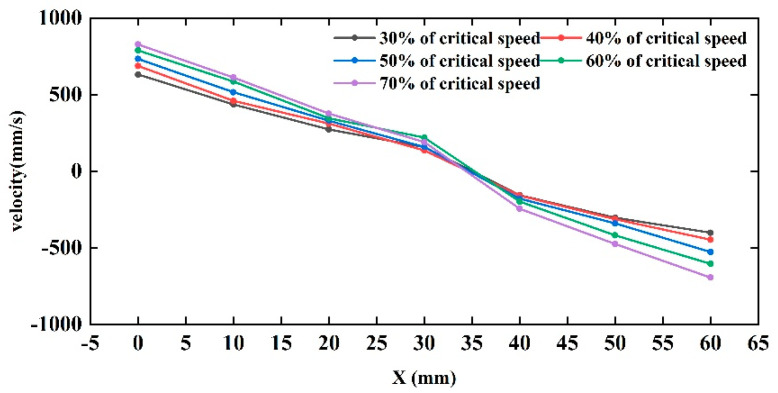
Velocity distribution along the center chord direction in the cylinder.

Figure 7a–e shows the velocity field distribution of the particles inside the cylinder at different rotation speeds. In Figure 8, a comparative analysis of the velocity field distribution of the particle population at different speeds shows that the higher the speed, the more obvious the regional stratification of the velocity field of the particle population. As the speed increases, the area of the free layer near the surface and the hysteresis layer near the wall becomes larger and larger, and the area of the intersection layer in the central region is continuously compressed. At the same time, there is a significant increase in the velocity of the motion of the particles in the free layer.

To quantitatively represent the variation pattern of rice grain velocity in the indented cylinder separator, a coordinate system was established on the surface of the grain layer (Figure 7). The average velocity of the rice particles from the free surface to the cylindrical wall was extracted along the positive direction of the *X*-axis for different cylinder rotation rates (see Figure 8).

It can be seen from Figure 8 that the rice grains at the free surface and near the wall of the indented cylinder have a greater velocity at the same rotation rate. Looking along the free surface towards the cylinder wall, the rate of the change in speed remains the same as the distance from the free surface increases until the speed is less than zero. After the velocity is less than zero, the rate of the change in velocity increases significantly as the distance from the free surface increases. This indicates that the closer to the cylinder wall, the greater the difference in velocity between adjacent layers and the more violent the relative motion.

The velocity variation of the rice grains was consistent from the free surface to zero velocity at different rotation rates, and the curves overlapped, indicating that the influence of rotation rate was small. This indicates that, as the speed increases, the difference in velocity between adjacent layers becomes greater, making it easier for the rice grains to move relative to each other, resulting in more uniform mixing.

### 5.3. Influence of the Parameters on the Separation Efficiency

#### 5.3.1. Influence of Rotational Speed on the Distribution of Particle Escape Angle

**Figure 9:**
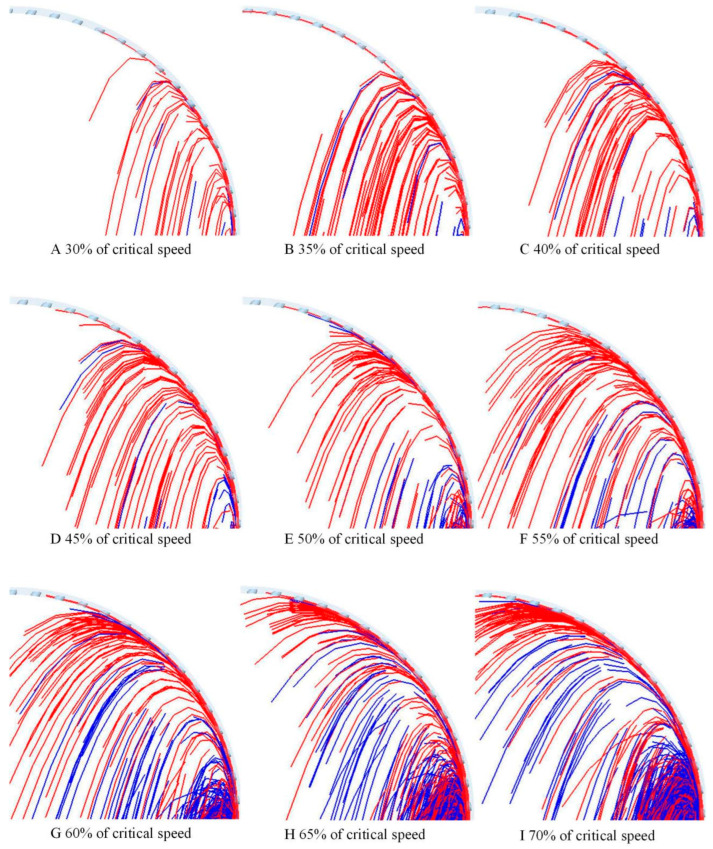
Motion trajectory of the whole rice (blue) and broken rice (red) in the cylinder with different rotational speed ratios. (**A**–**I**) Movement trajectory of rice particles at 30%, 35%, 40%, 45%, 50%, 55%, 60%, 65%, 70% critical speed.

**Figure 10:**
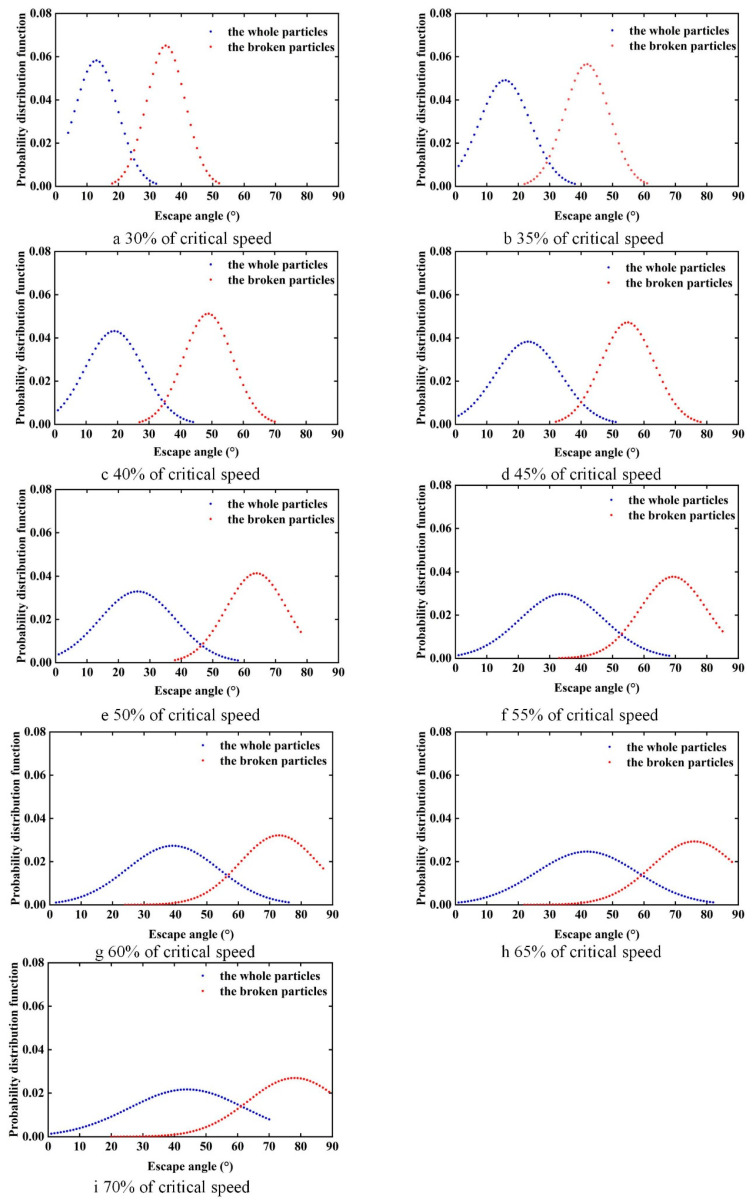
The probability distribution function of escape angle under various rotational speed ratio. (**a**–**i**) Escape angle probability distribution function at 30%, 35%, 40%, 45%, 50%, 55%, 60%, 65%, 70% critical speed.

To clearly compare the effect of the rotational speed on the particle separation process, the trajectories of the particles with different integrity in the first quadrant after mixing uniformly were selected, as shown in Figure 9. To quantitatively evaluate the difference between the separation angle of the whole and broken rice grains, the probability distribution function of the separation angle of rice grains at different rotational speeds was measured, as shown in Figure 10.

Figure 9 shows the trajectories of two types of rice grains at different rotation rates, with blue representing the whole rice grains and red representing broken rice grains. It is clearly shown that the angle of detachment of both whole and broken rice grains increases as the rotation rate increases and that the angle of detachment of broken grains is greater than that of the whole grains.

**Figure 11:**
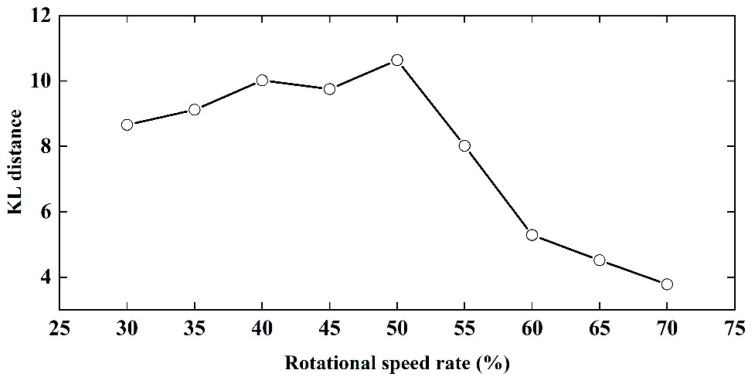
Variation of the KL distance with rotational speed ratio.

Figure 10 shows the probability distribution functions of the detachment angles of the two rice grains at different rotation rates. It can be seen from the graph that the probability distribution of the escape angle distribution of rice grains with different degrees of integrity shows a normal distribution trend. The mean value of escape angle distribution for both the whole and broken rice grains increased as the rotation rate increased. The difference between the mean values of the escape angle distributions of the different grains also changes with the increase in the rotation rate. The difference between the mean values of the escape angle distributions of the grains is one of the key parameters of cylinder separation: the larger the difference, the better the separating. The differences between the mean value of the escape angle of whole and broken rice particles increases and then decreases, and the range of the separating interval increases and then decreases. The differences in the probability distributions at different drum rates cannot be visualized from the graphs and the KL distance is used to assess the differences quantitatively and visually in the probability distribution function (Figure 11).

Figure 11 shows the KL distance statistics of the probability distribution function of the escape angle for intact and broken rice particles at different rotational speed rates. The larger the KL distance, the smaller the similarity of the distribution function. KL distance increases and then decreases as the speed increases from 30% to 70% and reaches a peak at 50%, implying that the similarity between the probability distribution of the separation angle of intact and broken rice particles decreases and then increases. In conclusion, 50% of the critical speed is the most favorable cylinder speed for sorting.

#### 5.3.2. Influence of Cylinder Axial Inclination Angle on the Distribution of the Particles’ Escape Angles

**Figure 12:**
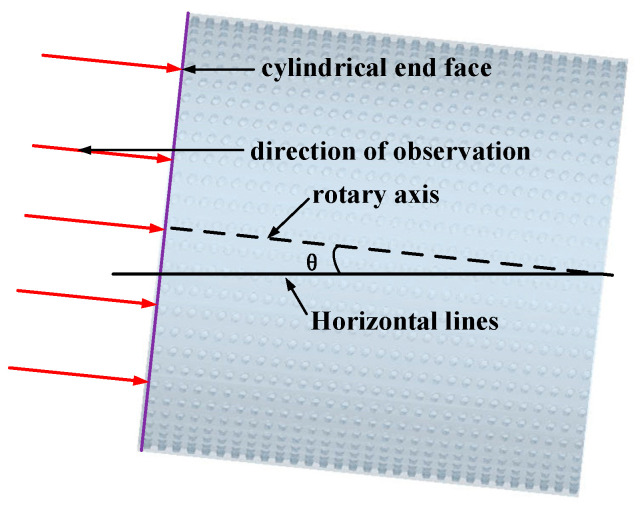
Diagram of inclined cylinder.

In the actual operation of the indented cylinders, the cylinder is placed at an incline to bring the rice particles inside the cylinder to the next stage, and the movement of the particles inside the inclined cylinder is different from that of a horizontally placed cylinder. To address this situation, this paper presents a numerical simulation of the movement of the cylinder at different inclinations. Figure 12 shows a schematic diagram of an inclined cylinder, with *θ* being the axial inclination angle of the cylinder and the red arrow showing the direction of the test observation.

**Figure 13:**
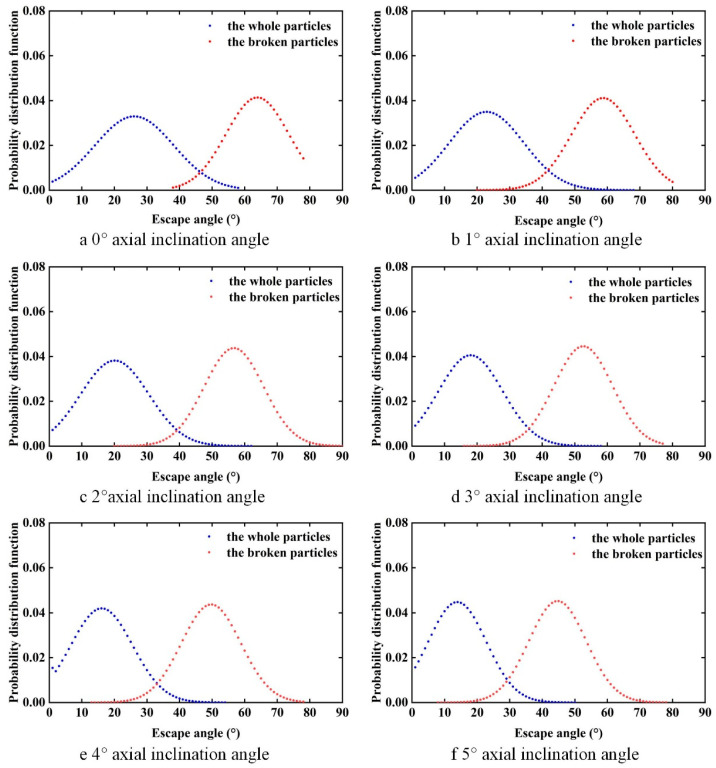
The probability distribution function of escape angle under various axial inclination angles. (**a**–**f**) Escape angle probability distribution function at 0°, 1°, 2°, 3°, 4°, 5° axial inclination angle.

To clearly compare the effect of different cylinder axial inclination angles on the particle separation process, the trajectories of rice particles inside the cylinder at different inclined angles after homogeneous mixing were selected. To quantitatively evaluate the difference between the escape angles of whole and broken rice particles, the probability distribution functions of the escape angles of rice particles inside the cylinders at different inclined angles were measured, as shown in Figure 13.

Figure 13 shows the probability distribution functions for the escape angles of the two rice grains at different cylinder axial inclination angles. It can be seen from the figure that the mean value of the distribution of escape angles for both whole and broken rice grains decrease with increasing inclination angle, and the difference in the mean value of the distribution of escape angles also varies with increasing inclination angle.

**Figure 14:**
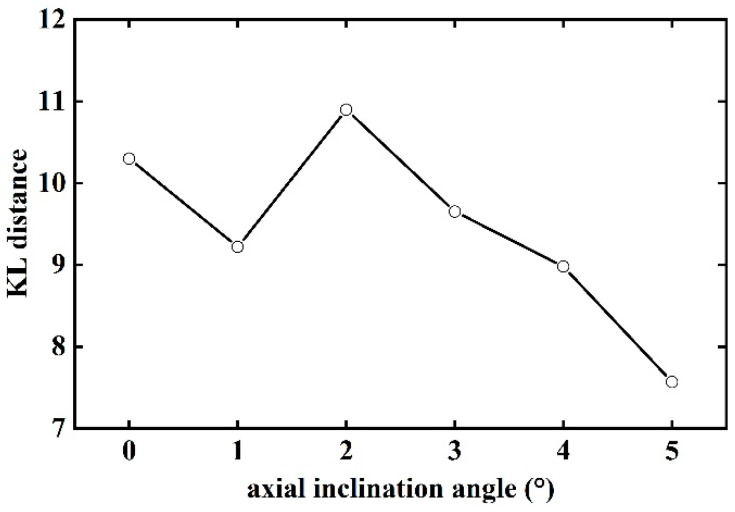
Variation of the KL distance with axial inclination angle.

The calculated KL distances as a function of axial inclination angle are summarized in Figure 14. The results show that the KL distance increases with increasing inclination angle until it reaches a maximum when the tilt angle is 2°. The KL distance starts to decrease at an even rate as the inclination angle increases further. The larger the KL distance, the better the particle separation, meaning better separating efficiency. The 2° angle of inclination is the best parameter obtained from the test.

#### 5.3.3. Inclination Angle of the Collection Trough

Using a 50% critical speed and the 2° cylinder inclination angle as test parameters, the critical escape angle for intact particles with a combined escape angle probability distribution of 0.95 was determined to be 37°, according to the obtained probability distribution diagram. A total of 95% of the whole rice particles had an escape angle distribution between (0,37°]. The rice particles are lifted as the cylinder rotates, gain a horizontal velocity, and make a parabolic motion when they leave the indents, and the trajectory of the particles after they leave the indents can be determined according to Equations (10) and (11).

(10) X=Rcosψ−Rωsinψt

(11) $$Y=R\sin\psi +R\omega \cos\psi t-\frac{1}{2}gt^{2}$$

where X is the horizontal coordinate of the particle. Y is the vertical coordinate of the particle. R is the cylinder radius. ω is the rotation speed. ψ is the escape angle of the particle. t is the movement time of the particle.

**Figure 15:**
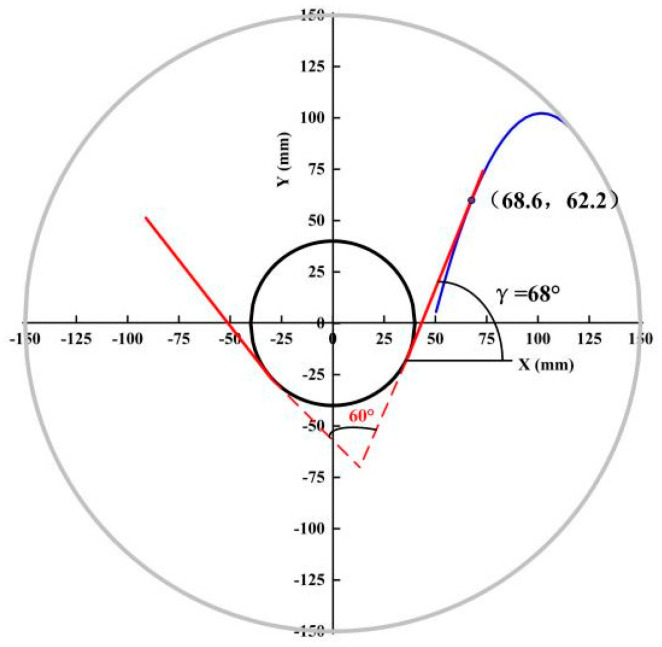
Optimum collection trough inclination angle.

As shown in Figure 15, the blue parabola shows the trajectory of the particle after it has broken away from the indents at an escape angle of 37°, and the black circle shows the circle in which the arc of the collecting trough is located. The angle between the red line and the *X*-axis is the theoretically optimum angle of inclination. The angle between the red line and the *X*-axis is the optimum inclination angle. Then the theoretically optimum angle of inclination of the collection trough the baffle can be obtained as 68°.

Separation efficiency is an important indicator of the performance of indented cylinders and can effectively reflect the degree of completion of the separating operation. Ideally, when a mixture of intact and broken particles enters the indented cylinder separator, the broken particles are lifted to a disengaged position and fall into the collection trough, while the whole particles return to the bottom of the indented cylinder. In the actual separating process, it is not possible to achieve the ideal separating condition, as some of the broken rice particles will fall out of the indented cylinder at a lower position and not enter the collecting trough, resulting in incomplete separating.

In this paper, the ratio of the number of broken rice particles in the collecting trough to the total number of broken rice particles in the sample is used as the separation efficiency of the indented cylinder, and the ratio of the number of whole rice particles in the collecting trough to the total number of whole rice particles in the sample is used as the loss ratio of the indented cylinder. The separation efficiency and loss rate are used as evaluation indicators to evaluate the separating effect of the indented cylinder separators and provide reference for achieving optimal separating.

The separation efficiency and loss rate can be obtained according to Equations (12) and (13).

(12) $$SE=\frac{NB}{TB}$$

(13) $$LR=\frac{NW}{TW}$$

where SE is the separation efficiency of the indented cylinder separator. NB is the number of broken rice particles in the collection trough. TB is the total number of broken rice particles in the sample. LR is the loss ratio of the indented cylinder separator. NW is the number of the whole rice particles in the collection trough. TW is the total number of the whole rice particles in the sample.

**Figure 16:**
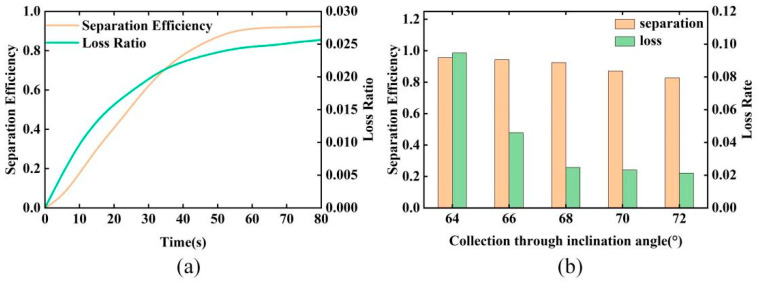
(**a**) Plot of the separation efficiency and loss rate over time. (**b**) Variation of separation efficiency and loss ratio with time for different collection trough inclinations.

In order to verify the accuracy of the theoretical optimum baffle inclination angle obtained and the effect of the size of the collection trough inclination angle on the separation efficiency, tests were carried out at 50% speed, 2° of cylinder axial inclination, and different collection trough inclination angles, as shown in Figure 16.

As can be seen from Figure 16a, at the optimum speed and axial inclination angle of the cylinder, the separation efficiency increases rapidly and then slowly with time, with decreasing growth and after 60 s, the separation efficiency remains almost stable and reaches the maximum separation efficiency. The trend of loss rate with cylinder movement time was like that of separation efficiency over time.

Figure 16b shows the statistical graphs of separation efficiency and loss rate near equilibrium for different collection trough baffle inclination angles. Both the separation efficiency and loss rate showed a decreasing trend with the increase of the collection trough baffle inclination angle. The separation efficiency decreases slowly with the increase of the collection chute baffle inclination angle, and when the collection chute inclination angle exceeds 68°, the separation efficiency starts to decrease more significantly. The loss rate decreases rapidly with the increase of the collection chute baffle inclination angle and decreases slightly when the baffle tilt angle exceeds 68°.

## 6. Conclusions

A model of whole rice particles and broken rice particles was established by the discrete element method, and the movement process of rice particles in the indented cylinder was simulated and analyzed. The probability distribution functions of the escape angles of particles with different rotation rate of cylinder, cylinder axial inclination angles, and collection trough inclination angles were studied, and the KL distance was used to quantitatively evaluate their separation capacity, and the working parameters with the best separation efficiency were derived.

The key findings are as follows.

As the cylinder rotation rate increases, the escape angle of both intact and broken rice grains increases. At the same time, the escape angle distribution range becomes larger, the overlapping area of the escape angle distribution range decreases and then increases, the KL distance increases and then decreases, and the maximum value is obtained at 50% of critical speed, which corresponds to the theoretically optimum separation speed.

With the increase of the axial inclination angle of the cylinder, the escape angle of both intact and broken rice kernels decreases. At the same time, the KL distance first decreases, then increases and then decreases again, reaching a maximum at an axial inclination angle of 2°.

The optimum combination of parameters for the indented cylinder separator was calculated with a cylinder speed of 50% of the critical speed, an axial inclination angle of the cylinder of 2°, an inclination angle of the collection chute of 68°, a separation efficiency of 88.05%, and a loss rate of 2.92%.

## Figures and Tables

**Table 1:** Particle property parameters.

Items	Parameter	Value
Rice particle	Density ρ/(kg·m^−3^)	1350
	Poisson ratio μ	0.3
	Shear modulus G/Pa	2.6 × 10^6^
Cylinder	Density ρ/(kg·m^−3^)	7800
	Poisson ratio μ	0.29
	Shear modulus G/Pa	77.2 × 10^9^
Particle–particle	Restitution coefficient	0.6
	Coefficient of static friction	0.5
	Coefficient of rolling friction	0.01
Particle–cylinder	Restitution coefficient	0.5
	Coefficient of static friction	0.56
	Coefficient of rolling friction	0.01

## Data Availability

On behalf of our team, we declare that all the data and material in this research is available.
